# A Novel Obligate Intracellular Gamma-Proteobacterium Associated with Ixodid Ticks, *Diplorickettsia massiliensis*, Gen. Nov., Sp. Nov

**DOI:** 10.1371/journal.pone.0011478

**Published:** 2010-07-13

**Authors:** Oleg Mediannikov, Zuzana Sekeyová, Marie-Laure Birg, Didier Raoult

**Affiliations:** 1 Unité des Recherches sur les Maladies Infectieuses et Emergentes, Centre National de la Recherche Scientifique 6236, Institut de Recherche pour le Développement 198, Université de la Méditerranée, Marseille, France; 2 Institute of Virology, Slovak Academy of Sciences, Bratislava, Slovak Republic; Duke University Medical Center, United States of America

## Abstract

**Background:**

Obligate intracellular bacteria of arthropods often exhibit a significant role in either human health or arthropod ecology.

**Methodology/Principal Findings:**

An obligate intracellular gamma-proteobacterium was isolated from the actively questing hard tick *Ixodes ricinus* using mammalian and amphibian cell lines. Transmission electron microscopy revealed a unique morphology of the bacterium, including intravacuolar localization of bacteria grouped predominantly in pairs and internal structures composed of electron-dense crystal-like structures and regular multilayer sheath-like structures. The isolate 20B was characterized to determine its taxonomic position using a polyphasic approach. Comparative 16S rRNA gene sequence analysis showed that this strain belongs to the family *Coxiellaceae*, order *Legionellales* of Gamma-proteobacteria, and the closest relatives are different *Rickettsiella* spp. The level of 16S rRNA gene sequence similarity between strain 20B and other recognized species of the family was below 94.5%. Partial sequences of the *rpoB*, *parC* and *ftsY* genes confirmed the phylogenetic position of the new isolate. The G+C content estimated on the basis of whole genome analysis of strain 20B was 37.88%. On the basis of its phenotypic and genotypic properties, together with phylogenetic distinctiveness, we propose that strain 20B to be classified in the new genus *Diplorickettsia* as the type strain of a novel species named *Diplorickettsia massiliensis* sp. nov.

**Conclusions/Significance:**

Considering the source of its isolation (hard tick, often biting humans) the role of this bacterium in the pathology of humans, animals and ticks should be further investigated.

## Introduction

Gamma-proteobacteria from the order *Legionellales* [1] are widespread in nature and often associated with a eukaryotic host. The order contains two taxonomic families, *Legionellaceae* and *Coxiellaceae*. All *Legionella* spp. in a monotypic family are potentially pathogenic for humans, although *L. pneumophila* plays the most important role in pathogenesis. These bacteria are often associated with amoebae [2]. The *Coxiellaceae* family is currently considered to include the two genera: *Coxiella* and *Rickettsiella*. *Coxiella* comprises only one species, *C. burnetii*, which is an emerging pathogen of humans and animals. *C. burnetii* is sometimes associated with arthropod hosts, often with Ixodid and Argasid ticks [3]. To date, all bacteria of this genus belong to one species; however, genetic variation within the species divides strains into multiple genogroups [4]. Nevertheless, genes of *Coxiella*-like bacteria that are genetically similar to *C. burnetii* have been repeatedly amplified from different sources, mostly hard and soft ticks [5], [6], but never isolated.

The genus *Rickettsiella* comprises intracellular bacteria associated with a wide range of different arthropods (insects, arachnids, isopods) that are currently classified into the three recognized species: *Rickettsiella popilliae*, *Rickettsiella grylli*, and *Rickettsiella chironomi* [7]. Most of our knowledge about *Rickettsiella* is based on light and electron microscopic observations, and bacteria were initially classified in the genus *Rickettsiella* mainly on the basis of morphologic criteria, including their intracellular location, their oval or rod-like to pleomorphic forms, the occurrence of a complex intravacuolar cycle and the occurrence of crystalline-like structures. There is no laboratory strain in axenic media or cell culture currently available.

*Rickettsiella* spp. are considered pathogens of arthropods, but in many cases, asymptomatic carriership is found. Until recently, bacteria from the genus *Rickettsiella* were thought to be close to rickettsiae and were even called “rickettsiae of insects”. However, phylogenetic studies showed that, despite their intracellular localization, they belong to gamma-proteobacteria, close to the genera *Legionella* and *Coxiella* [8]. Pathogenicity to a vertebrate host via inhalation has been shown using a mouse experimental model [9], [10]. However, studies of host specificity, attempts at cultivation in cell or axenic media, and antigenic analyses have not yet been very extensive or successful. To the best of our knowledge, no continually sustained cell culture of rickettsiellae exists, and the only method of cultivation is in laboratory insect lines. The genome of *R. grylli* is published in GenBank (accession number AAQJ00000000), though annotation was generated automatically without manual curation. Based on the analysis of several genes, a separate taxonomic position was proposed for rickettsiellae [11].

Ixodid ticks are obligate hematophagous arthropods that parasitize vertebrates, and bacteria that are phylogenetically close to the genus *Rickettsiella* were once observed in ticks of genus *Ixodes* [12]. *Ixodes ricinus*, also called European sheep tick, is the most prevalent and widely distributed tick species in Central Europe, particularly in the Alpine Region. It is also the tick species that most frequently bites humans in Europe [13]. The bacteria and viruses associated with this tick are quite well studied, because of its importance for human and animal health. Almost all bacteria isolated from *I. ricinus* are pathogenic for humans. This tick is known to harbor microorganisms such as *Borrelia* spp. (*B. burgdorferi, B. afzelii,* and *B. garinii*), *Rickettsia helvetica*, *Rickettsia monacensis, Anaplasma phagocytophilum*, *Ehrlichia* spp., and *Francisella tularensis,* as well as a number of viruses and protozoa; moreover, the tick is known to transmit such pathogens to humans and animals [14]. Control of tick populations is one of the most important factors for reducing morbidity by tick-borne diseases [15]; acaricides continue to be the most effective and widely used approach for tick control, although biological approaches show benefits as well.

The present study aimed to identify other obligate intracellular bacteria associated with such hematophagous arthropods as hard ticks and describe its morphology, and phylogeny.

## Materials and Methods

### Isolation and light microscopy

Adult *Ixodes ricinus* ticks were collected with a standard muslin drag in the southeastern part of the Slovak republic forest Rovinka in 2006. The total number of ticks prepared for strain isolation counted 36. Ticks were washed with distilled water, sterilized by immersion in iodinized alcohol and rinsed in distilled water. Initial isolation was done in a mouse fibroblast cell line L929 (ATCC® number CCL-1™) at 34°C using the standard shell vial technique [16]. Minimal essential medium (MEM) (Invitrogen, Cergy-Pontoise, France) supplemented with 4% fetal bovine serum (FBS) (Invitrogen) and 1% L-glutamine (Invitrogen) was used for cultivation. Cells were maintained in a 5% CO_2_ atmosphere at 35°C. The cultures were analyzed once a week for the presence of intracellular bacteria by Gimenez staining [17]. Other cell lines were used to achieve a better bacteria accumulation, including XTC-2 (from *Xenopus laevis*) [18], HEL (ATCC® number CCL-137™) and MRC-5 [19] (both of human origin). Re-inoculation was accomplished by the shell-vial technique. Minimum Essential Medium Eagle supplied with 5% FBS was used for HEL and MRC-5. Leibovitz-15 (Invitrogen) supplemented with 2% of FBS and 2% of tryptose-phosphate broth solution (Sigma-Aldrich, Ayrshire, UK) was used for XTC-2 cell lines.

Cultivation attempts on axenic media were done with Trypcase soy Agar, Columbia agar supplemented with 5% sheep blood (BioMérieux, Marcy l'Etoile, France), Legionella BCYE medium and Legionella BHPA selective medium (Oxoid, Wesel, Germany).

In 2010, another collection of *I. ricinus* ticks was performed in the same forest, in total 80 ticks (28 adults and 52 nymphs).

### Electron microscopy

Infected cells were washed with PBS and fixed overnight in 2% glutaraldehyde in 0.1 M cacodylate buffer. After being washed in 0.1 M cacodylate buffer, the specimens were post-fixed in 1% osmium tetroxide in 0.1 M potassium ferricyanide for 1 h and dehydrated in an ascending series of ethanol concentrations ranging from 30% to 100%. After absolute ethanol, dehydration was finished in propylene oxide. The samples were embedded in Epon 812 resin. Sections (70 nm) were stained with 5% uranyl acetate and lead citrate before examination in a transmission electron microscope (Philips Morgagni 268D). For better visualization of the carbohydrate layer, another series was treated by ruthenium red.

Negative staining for transmission electron microscopy was performed as follows. The infected cells were gently collected in their culture medium under a biosafety cabinet. A first centrifugation was performed at 168 g for 10 min to separate the cells (both infected and uninfected) from the free bacteria burst from over-infected cells in a medium. A drop of the supernatant was collected on a piece of parafilm. A nickel 400 mesh Formvar carbon grid was deposited on top of the drop for 10 min at 37°C. The contrast used was ammonium molybdate 1% for 10 sec. Before being removed from the PSM grid, they were exposed to UV for 1 hour.

### PCR amplification, sequencing and phylogenetic analysis

Bacterial DNA was extracted from the supernatant of infected cells using the Qiamp DNA MiniKit according to the manufacturer's instructions (Qiagen)**.** DNA from ticks for epidemiological studies was extracted as reported previously [5].

Universal eubacterial primers were used for amplification of almost the complete 16S ribosomal RNA gene [20]. The *rpoB* and *tuf* genes were chosen for amplification because they are useful tools for the molecular characterization of many bacterial species, including a number of gamma-proteobacteria. A pair of primers, RLrpoB6f (5′-AGATGGTACGCSGGTTGATATCGT-3′) and RLrpoB2r (5′-TTCCATTTGGTGATCGCCATC-3′), was designed based on conserved regions of the *rpoB* gene of different representatives of the *Legionellales* order. Analogously, based on conserved regions, a set of primers was designed to amplify a portion of the *tuf* gene of gamma-proteobacteria: RLtuf1f (5′-TTAGGCCTTTTTACCTTTTT-3′) and RLtuf3r (5′-GGCCACTGGAAAGTTTGTGCG-3′). Two pairs of primers were designed for the amplification of almost the complete *ftsY* gene sequence of the isolated bacterium, DM_ftsY-2f→5′-TTACTGAAAACACGCCATCA-3′ with DM_ftsY-10r→5′-TTACCAATCGTCGTCGTCTTC-3′, and DM_ftsY-9f→5′-GAAGACGACGACGATTGGTAA-3′ with DM_ftsY-8r→5′-TCCTCCTTTGGCAGTCCCATC-3′.

A qPCR system for *rpoB* gene of strain 20B was designed, its specificity and sensitivity was tested as reported before [21]. Primer sequences are: 1Diplo_rpoB_L CCGCGCAAAAAGTTGATCT; 1Diplo_rpoB_R GTAAGTCCGCAAGACGAAGC; probe 6′FAM-CCAGACAATGAGTTGCTCGA-TAMRA.

The phylogenetic tree was calculated by using the neighbor-joining method with MEGA3 software (The Biodesign Institute, Tempe, Arizona, USA). Evolutionary distances for the defined groups were calculated using the Tamura-Nei parameter model. Internal node support was verified using the bootstrap method with 100 replicates. The sequences of the *rrs* (16S rDNA) gene used for comparison were obtained from the GenBank database (http://www.ncbi.nlm.nih.gov) based on the closest similarities with the sequence of *Diplorickettsia massiliensis* sp. nov. Sequences were aligned and corrected manually for conserved motifs. Sites with ambiguous alignments were removed before phylogenetic analysis.

### Results and Discussion

**Figure 1:**
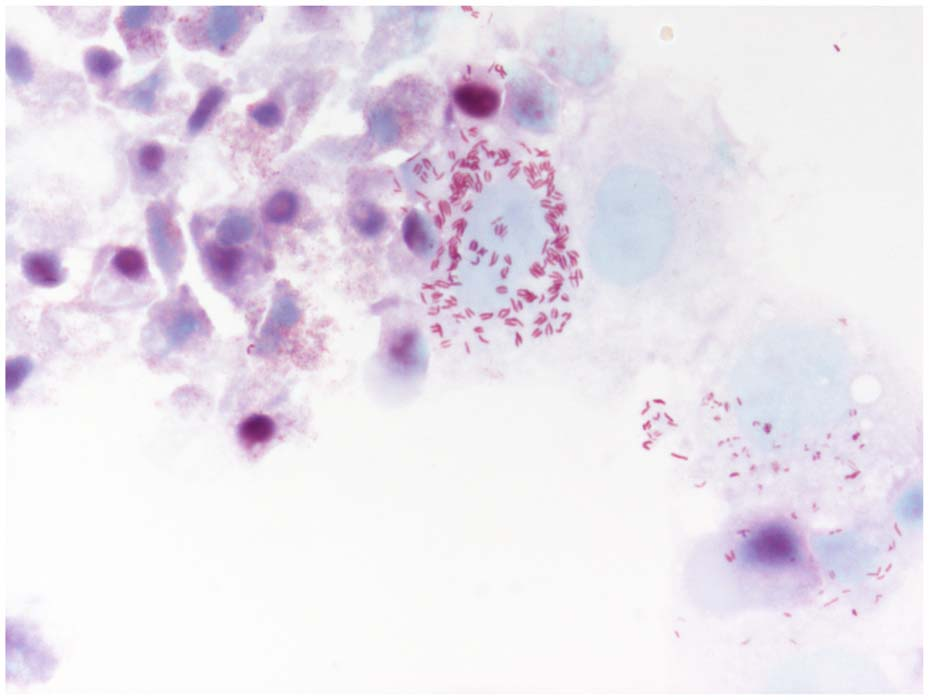
Strain 20B grown in XTC-2 cells, Gimenez staining, x1500.

We have succeeded in the isolation of a novel intracellular bacterial strain (20B) from one *I. ricinus* tick of 36 studied. Bacteria visualized in a rich culture established in XTC-2cell line by Gimenez staining appeared as intracellular red rods usually grouped in pairs but not connected with each other (Figure 1). Manual counting of bacteria in an unlysed eukaryotic cell showed that almost all bacteria (97%) were paired. The bacteria accumulated in the cytoplasm of cells but not in the nucleus. The maximum number of bacteria observed in one cell numbered over 100. Infected cells were often disrupted during centrifugation using a Cytospin (Thermo Shandon) centrifuge as revealed by subsequent staining. The isolate was Gram-negative when extracellular bacteria were stained.

In addition, we have examined the percentage of infected cells and the mean number of bacteria per cell in different cell lines. The highest growth speed and the presence of a cytopathogenic effect when bacteria were cultivated in XTC-2 cells (*Xenopus laevis*). A cytopathogenic effect, including cellular layer detachment and cell disruption, was observed 3–5 days after inoculation. It was the only cell line with 100% cells infected the mean number of bacteria per cell was more then 100 in all studied series. The growth speed and bacteria accumulation in cells were lower in cell lines of human origin (HEL and MRC5) cultivated at 32°C but were minimal in mouse L929 cells (data not shown).

Multiple attempts at cultivation of the bacteria in solid axenic media (both common and *Legionella*-specific) were not successful.

Search of bacterial DNA by qPCR in *I. ricinus* ticks collected 3 years later in the same forest did not yielded positive results.

### Morphology by electron microscopy

**Figure 2:**
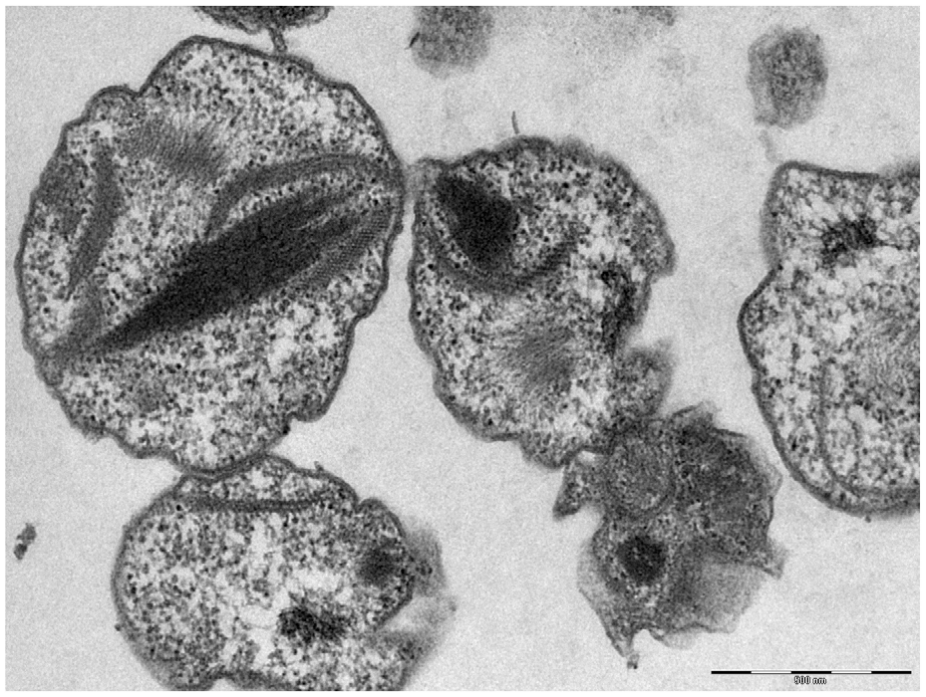
Strain 20B bacteria grown in XTC-2 cells Transmission electron microscopy; staining with red ruthenium. The absence of a glycoprotein surface layer.

Negative staining showed that the bacteria have an average length of 1540 nm (range: 848 to 3067 nm) and an average diameter of 695 nm (range: 515 to 992 nm). It should be noted that the longest bacteria were in the process of division. The bacteria of the strain 20B were harvested when they were extracellular for the studies of surface structures. Unlike other intracellular bacteria [22], including rickettsiae, 20B strain failed to highlight surface glycoproteins when colored with ruthenium red (Figure 2).

**Figure 3:**
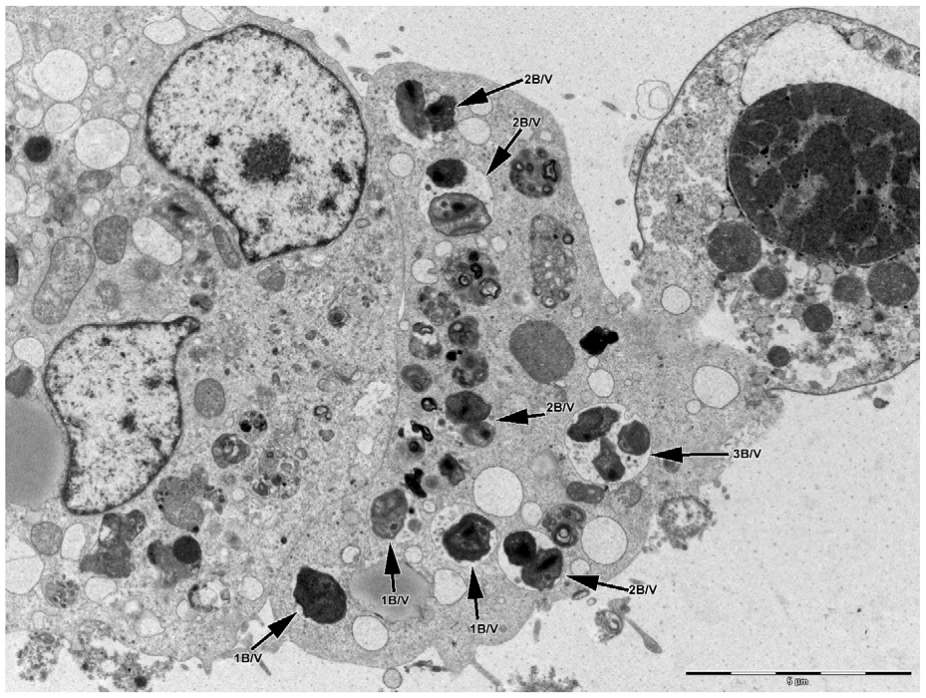
Strain 20B bacteria grown in XTC-2 cells. Transmission electron microscopy; staining with uranyl acetate. The number of bacteria per vacuole (B/v) is indicated by arrows.

**Figure 4:**
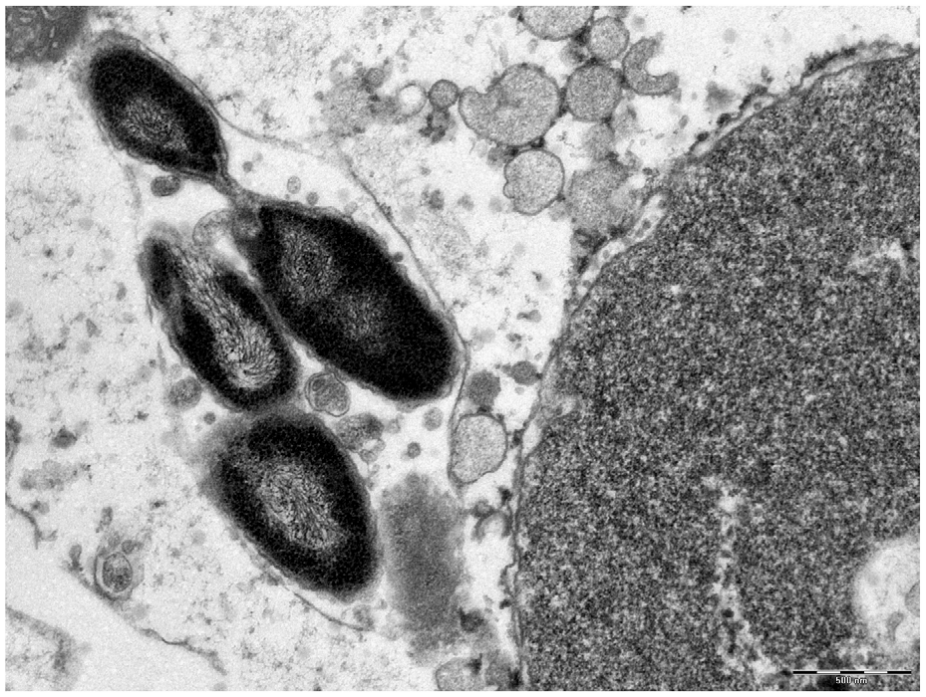
Strain 20B bacteria grown in XTC-2 cells Transmission electron microscopy; staining with uranyl acetate. The bacteria in the process of division.

All bacteria observed intracellularly were located in vacuoles. Unlike rickettsiellae, they do not present a regular organization suggestive of crystalline structure [12], [23]. Based on our visual impression of paired bacteria (Figure 1), we have counted the number of bacteria in vacuoles across the section: 51.4% of vacuoles contained 2 bacteria, 13.2% contained 3 or 4 bacteria, 1.7% contained more than 4, and 33.7% contained 1 bacterium (Figure 3). The ultrathin section may pass across only one bacterium in a vacuole that actually contains multiple bacteria, so this may mean that a number of these pseudo-single bacteria may be also paired. Taking these data into consideration, we came to the conclusion that most bacteria are paired inside vacuoles. We also found bacteria in the process of division within the vacuoles (Figure 4).

**Figure 5:**
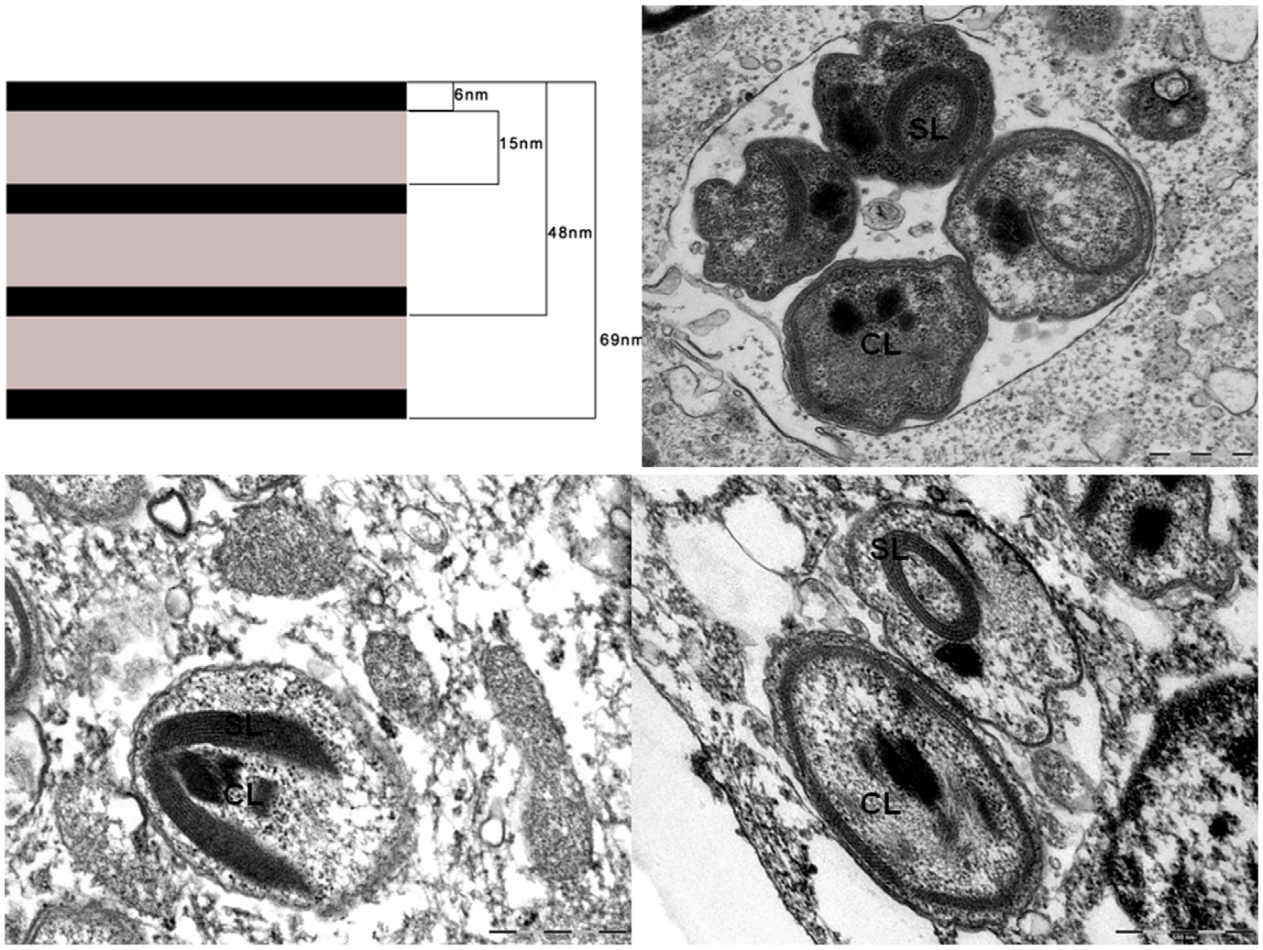
Strain 20B bacteria. Transmission electron microscopy; staining with uranyl acetate. The internal bacterial structure: electron-dense crystal-like (CL) structures located in the center of almost all bacteria. Multilayer sheath-like (SL) structure are alternating electron dense (6 nanometers), electron light (15 nanometers) layers (upper left).

The internal structure of the bacteria was atypical (Figure 5). We have identified electron-dense crystal-like structures located in the center of almost all bacteria, usually surrounded by multilayer sheath-like structures. These layers alternate with electron-dense bands (6 nanometers) and light bands (15 nanometers) (Figure 5). Up to seven electron-dense layers may be found in a single bacterium.

### Estimation of the G+C content

Determination of the G+C content of prokaryotic genomes using traditional methods is time-consuming, and results may vary from laboratory to laboratory, depending on the technique used. Furthermore, recent studies have shown that conventional evaluation of the G+C content of the bacterial genome is not always suitable for intracellular bacteria with a low G+C content [24], [25]. However, the genomic G+C content of prokaryotes may also be estimated from the sequence of the *ftsY* gene, which codes for the cell division membrane protein [24]. This gene is highly conserved (median size 1144 nucleotides) and is a vertically inherited member of the GTPase superfamily.

We succeeded in amplifying and sequencing a nearly full-length 724-bp portion of the *ftsY* gene from the isolated bacterium using degenerate primers located at the ends of the gene. The calculated G+C content of this portion was 43.55%.

At the same time, we have begun to sequence the whole genome of this bacterium. Based on our genome sequencing of the entire *ftsY* gene, this gene showed a G+C content of 42.14%. Meanwhile, the G+C content of the whole genome was 37.88% (data to be published separately).

### Phylogenetic analysis

**Figure 6:**
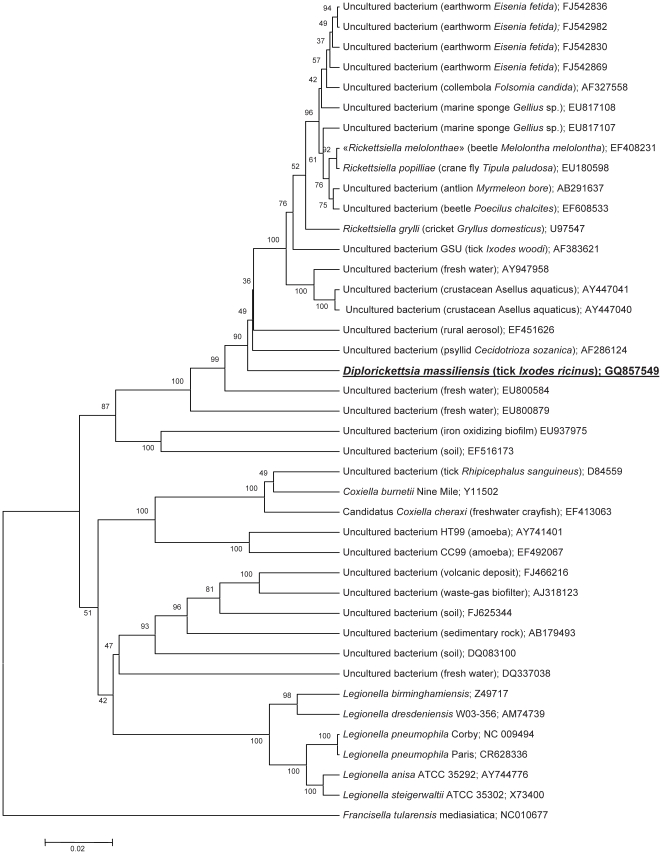
Phylogenetic tree based on aligned complete sequences of the *rrs* (16SrRNA) gene and constructed by the UPGMA method. The numbers in the nodes represent bootstrap values. The tree shows the position of *Diplorickettsia massiliensis* (isolate 20B) among recognized bacterial species and *rrs* genes amplified from uncultured bacteria and environmental samples (Genbank). The host (if any) or the source of amplification is indicated in the brackets; GenBank accession number is indicated at the end of the line for each sequence.

We succeeded in amplifying and sequencing a 1476-bp fragment of the *rrs* gene encoding the 16S ribosomal RNA. Surprisingly, a BLAST search did not result in a close similarity with any published 16S RNA gene sequences from other bacteria. The closest identity found was less than 94.5% and was with different representatives of the *Rickettsiella* genus (the closest among validated species is *Rickettsiella popiliae*) and multiple uncultured bacteria derived from environmental samples. Consequently, isolate 20B represents a separate species according to the practical 3% cut-off value for 16S rRNA divergence to demarcate species [26]. The phylogenetic tree (Figure 6) based on this gene shows that this isolate groups with other bacteria of the order *Legionellales* [1] of gamma-proteobacteria. The closest bacterium grouped with isolate 20B was an uncultured eubacterium (a secondary endosymbiont) from a psyllid *Cecidotrioza sosanica* [27].

Using primers designed for the *rpoB* gene, a 729-bp amplicon was obtained by PCR and sequenced. In a BLAST search, the unique 68-bp sequence showed no complete homology with any microorganism. However, different degrees of identity (up to 84%) of a conserved section of 184–280 bp were found with *rpoB* genes of *Proteobacteria*, including gamma-proteobacteria.

Surprisingly, a 556-bp fragment amplified by PCR with primers designed to amplify the *tuf* gene showed homology with another gamma-proteobacterial gene, *parC*, which codes for the DNA topoisomerase IV subunit A. It exhibited a maximum identity of 84% with *Haemophilus somnus* (GenBank accession number CP000947.1). The degree of identity with *Legionella pneumophila* (AE017354.1) was 70%. The amplification of the *parC* gene instead of *tuf*, for which the primers were designed, may be explained by occasional homology of designed primers.

The 724-bp portion of the *ftsY* gene also showed the absence of complete homology with any other bacterium, although the 5′- portion (62% of the amplified gene) displayed 70% homology with the *ftsY* gene of the *Legionella pneumophila* Corby strain (accession number CP000675.2).

### Taxonomic conclusions

Polyphasic approaches, including unique morphology (Figures 1, 2, 3, 4, 5) and 16S rDNA phylogenetic analyses (Figure 6) support the placement of the 20B isolate into a novel genus within the order *Legionellales* and the family *Coxiellaceae*.

The absence of any isolates in cell culture of the closest genus *Rickettsiella*, as well as 20B's intracellular lifestyle, prevents comparison of biochemical characteristics of this isolate with other phylogenetically associated species. The unique internal bacterial structure, the low 16S rDNA similarity (94%) with other representatives of the family, and its obligate intracellular lifestyle are evidence of the phylogenetic uniqueness of 20B. The G+C content varies from 36 to 41% in the *Rickettsiella* genus [28], and the identified 37.88% G+C content in isolate 20B also confirms the taxonomic position of this bacterium the *Legionellales*.

On the basis of both a phylogenetic and phenotypic distinction, we formally propose the *Diplorickettsia* gen. nov. to include the type species *Diplorickettsia massiliensis* sp. nov.

The tick *I. ricinus* is a notorious vector of multiple diseases of humans and animals. Many bacteria were first isolated from this tick and later found to be pathogenic, including *Rickettsia monacensis* [29] and *Rickettsia helvetica* [30]. The role of the bacterium isolated in this study with respect to human and animal health remains to be evaluated, although obligate intracellular bacteria that successfully grow in human cells may easily be pathogenic. We did not find *D. massiliensis* in *I. ricinus* ticks collected in the same forest 3 years after the isolation was performed. It may signify that the bacterium is either rare or may be the pathogenic for ticks and could not be easily found in a healthy tick population. The probable pathogenicity of this bacterium in ticks may also be of importance from the perspective of tick population control. Finally, if this bacterium is a mutualist/endosymbiont, its role in evolution and the tick lifecycle should be investigated.

### Description of *Diplorickettsia* gen. nov

*Diplorickettsia* (Di.plo.ri.ket̀.sia Gr. adj. diplos doubled; N.L. fem. n. *Rickettsia*, a bacterial generic name; N.L. fem. n. *Diplorickettsia,* doubled Rickettsia, for the phenotypic resemblance of the isolated strain with rickettsiae shown by Gimenez staining).

The cells are Gram-negative, non-spore-forming small rods. The bacteria are obligate intracellular and occur predominantly in pairs inside vacuoles of eukaryotic cells (tick, amphibian, and mammalian). The bacteria grow in XTC-2 cells at 28°C in Leibovitz-15 medium, supplemented with 2% heat-inactivated fetal calf serum, 2% tryptose-phosphate and 2 mM glutamine. The bacteria are non-motile, and 16S rRNA, *rpoB*, *parC* and *ftsY* gene sequencing indicate that this bacterium is clearly different from all other recognized species. The most closely related officially recognized species are *Rickettsiella* spp., and the type species is *Diplorickettsia massiliensis.*

### Description of *Diplorickettsia massiliensis* sp. nov

*Diplorickettsia massiliensis* (mas.si' li.en.sis. L. gen. adj. massiliensis, from Massilia, the Latin name of Marseille, France, where the organism was first grown, identified and characterized). The description is for that of genus type strain 20B. The known geographical distribution of this bacterium is Slovakia. This isolate has been deposited in the collection of the two World Health Organization Collaborative Centers for Rickettsial Reference and Research in Bratislava, Slovak Republic and the Faculté de Médecine, Université de la Méditerranée in Marseille, France (UR RIELLA NHT 117), as well as in the German Collection of Microorganisms and Cell Cultures (Deutsche Sammlung von Mikroorganismen und Zellkulturen, DSMZ) under the reference DSM 233381.

The GenBank/EMBL/DDBJ accession numbers for the 16S rRNA gene, *rpoB*, *parC* and *ftsY* sequences of strain B20 are GQ857549, GQ983049, GQ983050, and GU289825, respectively.

## References

[1] Garrity GM, Bell JA, Lilburn T, Brenner DJ, Krieger-Huber S, Stanley JT (2005). Order VI Legionellales.. Bergey's manual of systematic bacteriology.

[2] La Scola B, Mezi L, Weiller PJ, Raoult D (2001). Isolation of *Legionella anisa* using an amoebal coculture procedure.. J Clin Microbiol.

[3] Babudieri B (1959). Q fever: A zoonosis.. Adv Vet Sci Comp Med.

[4] Glazunova O, Roux V, Freylikman O, Sekeyova S, Fournous G (2005). *Coxiella burnetii* genotyping.. Emerg Infect Dis.

[5] Mediannikov OY, Ivanov LI, Nishikawa M, Saito R, Sidelnikov YN (2003). Molecular evidence of *Coxiella*-like microorganism harbored by *Haemaphysalis concinna* ticks in the Russian far east.. Rickettsiology: Present and Future Directions.

[6] Reeves WK, Loftis AD, Priestley RA, Wills W, Sanders F (2005). Molecular and biological characterization of a novel *Coxiella*-like agent from Carios capensis.. Ann N Y Acad Sci.

[7] Fournier PE, Raoult D, Brenner DJ, Krieger-Huber S, Stanley JT (2005). Genus II Rickettsiella.. Bergey's manual of systematic bacteriology.

[8] Roux V, Bergoin M, Lamaze N, Raoult D (1997). Reassessment of the taxonomic position of *Rickettsiella grylli*.. Int J Syst Bacteriol.

[9] Croizier G, Meynadier G (1972). Etude en immunofluorescence de l'infection expérimentale de la souris par *Rickettsia grylli*.. Ann Rech Vétér.

[10] Giroud P, Dumas N, Hurpin B (1958). Essais d'adaptation à la souris blanche de la rickettsie agent de la maladie bleue de *Melolontha melolontha* L.: voie pulmonaire et voie buccale.. C R Acad Science, Serie D.

[11] Leclerque A (2008). Whole genome-based assessment of the taxonomic position of the arthropod pathogenic bacterium *Rickettsiella grylli*.. FEMS Microbiol Lett.

[12] Kurtti TJ, Palmer AT, Oliver JHJ (2002). *Rickettsiella*-like bacteria in *Ixodes woodi* (Acari: Ixodidae).. J Med Entomol.

[13] Gern L (2005). The biology of the *Ixodes ricinus* tick.. Ther Umsch.

[14] Parola P, Raoult D (2001). Ticks and tickborne bacterial diseases in humans: an emerging infectious threat.. Clin Infect Dis.

[15] Piesman J (2006). Strategies for reducing the risk of Lyme borreliosis in North America.. Int J Med Microbiol.

[16] Kelly PJ, Raoult D, Mason PR (1991). Isolation of spotted fever group rickettsias from triturated ticks using a modification of the centrifugation-shell vial technique.. Trans R Soc Trop Med Hyg.

[17] Gimenez DF (1964). Staining rickettsiae in yolk-sac cultures.. Stain Technol.

[18] Pudney M, Varma MG, Leake CJ (1973). Establishment of a cell line (XTC-2) from the South African clawed toad, *Xenopus laevis*.. Experientia.

[19] Jacobs JP, Jones CM, Baille JP (1970). Characteristics of a human diploid cell designated MRC-5.. Nature.

[20] Weisburg WG, Barns SM, Pelletier DA, Lane DJ (1991). 16S ribosomal DNA amplification for phylogenetic study.. J Bacteriol.

[21] Mediannikov O, Fenollar F, Socolovschi C, Diatta G, Bassene H (2010). *Coxiella burnetii* in humans and ticks in rural Senegal.. PLoS Negl Trop Dis 6;.

[22] Waller LN, Fox N, Fox KF, Fox A, Price RL (2004). Ruthenium red staining for ultrastructural visualization of a glycoprotein layer surrounding the spore of *Bacillus anthracis* and *Bacillus subtilis*.. J Microbiol Methods.

[23] Louis C, Croizier G, Meynadier G (1977). Trame cristalline des inclusions proteiques chez une *Rickettsiella*.. Biol Cell.

[24] Fournier PE, Suhre K, Fournous G, Raoult D (2006). Estimation of prokaryote genomic DNA G+C content by sequencing universally conserved genes.. Int J Syst Evol Microbiol.

[25] Ezaki T, Saidi SM, Liu SL, Hashimoto Y, Yamamoto H (1990). Rapid procedure to determine the DNA base composition from small amounts of gram-positive bacteria.. FEMS Microbiol Lett.

[26] Stackebrandt E, Goebel BM (1994). A place for DNA-DNA reassorciation and 16S rRNA sequence analysis in the present species definition in bacteriology.. Int J Syst Bacteriol.

[27] Spaulding AW, von Dohlen CD (2001). Psyllid endosymbionts exhibit patterns of co-speciation with hosts and destabilizing substitutions in ribosomal RNA.. Insect Mol Biol.

[28] Frutos R, Federici BA, Revet B, Bergoin M (1994). Taxonomic studies of *Rickettsiella*, *Rickettsia*, and *Chlamydia* using genomic DNA.. J Invertebr Pathol.

[29] Jado I, Oteo JA, Aldamiz M, Gil H, Escudero R (2007). *Rickettsia monacensis* and human disease, Spain.. Emerg Infect Dis.

[30] Nielsen H, Fournier PE, Pedersen IS, Krarup H, Ejlertsen T (2004). Serological and molecular evidence of *Rickettsia helvetica* in Denmark.. Scand J Infect Dis.

